# Impact of climate change and seasonal trends on the fate of Arctic oil spills

**DOI:** 10.1007/s13280-017-0961-3

**Published:** 2017-10-24

**Authors:** Tor Nordam, Dorien A. E. Dunnebier, CJ Beegle-Krause, Mark Reed, Dag Slagstad

**Affiliations:** SINTEF Ocean, P. B. 4762 Torgard, 7465 Trondheim, Norway

**Keywords:** Arctic oil exploration, Climate change, Environmental risk assessment, Numerical simulations, Oil spill

## Abstract

We investigated the effects of a warmer climate, and seasonal trends, on the fate of oil spilled in the Arctic. Three well blowout scenarios, two shipping accidents and a pipeline rupture were considered. We used ensembles of numerical simulations, using the OSCAR oil spill model, with environmental data for the periods 2009–2012 and 2050–2053 (representing a warmer future) as inputs to the model. Future atmospheric forcing was based on the IPCC’s A1B scenario, with the ocean data generated by the hydrodynamic model SINMOD. We found differences in “typical” outcome of a spill in a warmer future compared to the present, mainly due to a longer season of open water. We have demonstrated that ice cover is extremely important for predicting the fate of an Arctic oil spill, and find that oil spills in a warming climate will in some cases result in greater areal coverage and shoreline exposure.

**Electronic supplementary material:**

The online version of this article (doi:10.1007/s13280-017-0961-3) contains supplementary material, which is available to authorized users.

## Introduction

Human activity in the Arctic (and elsewhere) is always associated with some risk of damage to the environment. In order to assess risk, one needs to estimate the probability of a given adverse outcome, as well as the consequences. In this paper, we study the probable outcomes of Arctic oil spills, using a selection of six different case studies. We specifically do not address the probability of a spill taking place, nor the consequences (i.e. the damage to natural resources), focusing instead on the transport and fate of the released oil, and the probability distributions associated with this fate.

We have studied the problem using ensembles of numerical simulations, which is a standard approach to investigating the possible outcomes of hypothetical oil spills (see e.g. Price et al. 2003; Guillen et al. 2004). Ensemble simulations are commonly used in the planning phase of new petroleum developments, and serve two goals: First, to provide the probabilities of for example beaching of oil, for a further Environmental Risk Assessment (ERA) where potential damage to natural resources will be considered, and second, to inform contingency planning for oil spill response, giving guidance on required amounts and distribution of response equipment (Barker and Healy 2001).

This study will investigate the effect of a future with a warmer climate on the footprint and fate of an Arctic oil spill. We consider not only average outcomes, but also the probability distribution of endpoints such as amount of beached oil (Nordam et al. 2016). The goal is first to determine if the “typical” transport and fate of an oil spill is different in a warmer climate, and second to determine if this has consequences for how Environmental Risk Assessments should be carried out in the Arctic, as we move towards a warmer future with more human activity in the High North.

## Materials and methods

The current study is carried out by numerical simulations, looking at a limited number of case studies selected to represent relevant oil spill scenarios that could occur in the Arctic. Below we present the numerical models used, as well as the scenarios studied.

### The SINMOD hydrodynamic model

Current and wind data are required as input for oil spill trajectory modelling. The SINMOD hydrodynamic model was used to produce the current data (Slagstad and McClimans 2005). SINMOD is based on the primitive Navier–Stokes equations and is established on a *z*-grid, using a constant-depth discretisation. The vertical turbulent mixing coefficient is calculated as a function of the Richardson number, *Ri*, and the wave state. The flow becomes turbulent when *Ri* is smaller than 0.65 (Price et al. 1986). Near the surface, vertical mixing due to wind waves is calculated from wind speed and fetch length. Horizontal mixing is calculated according to Smagorinsky (1963).

**Fig. 1:**
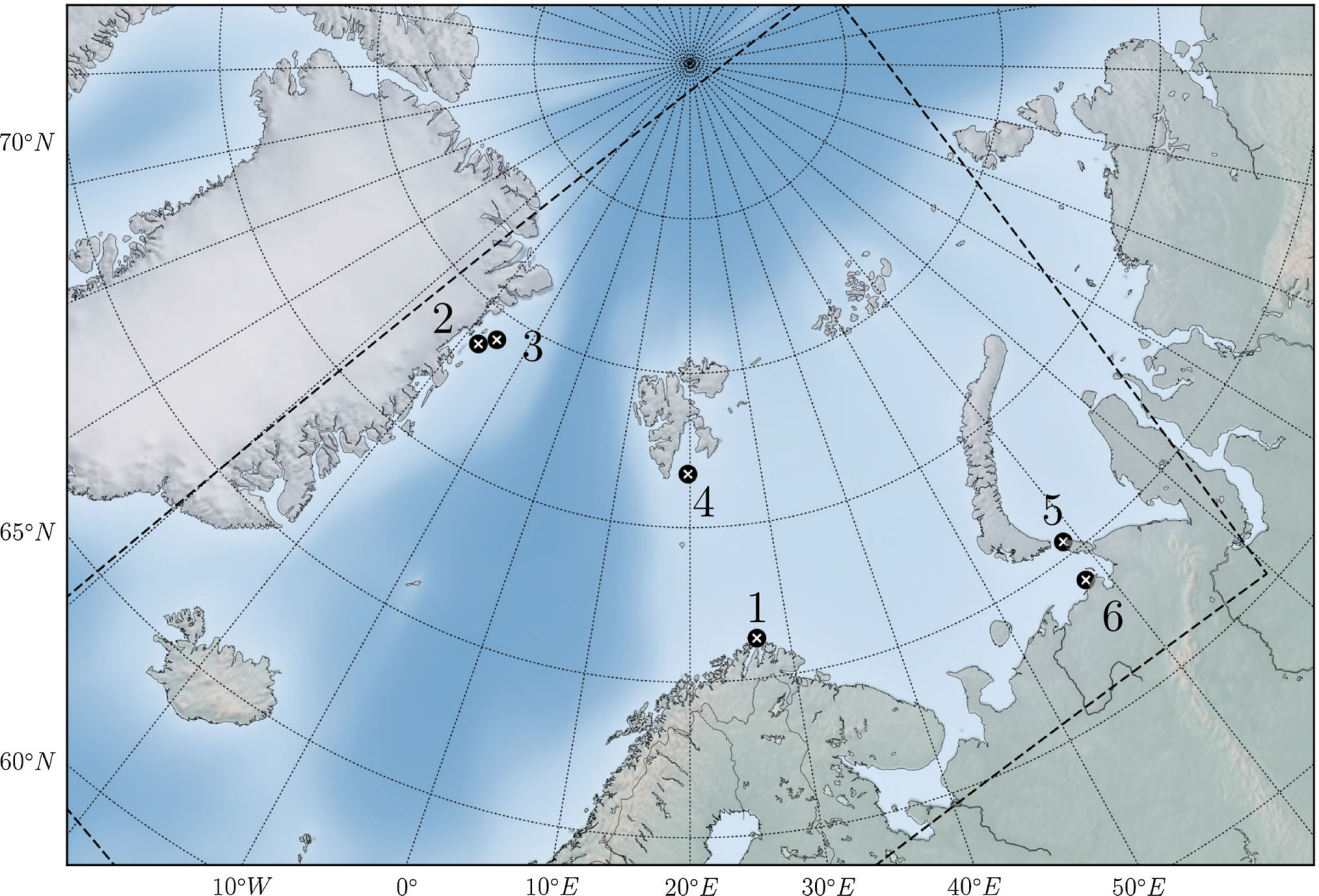
Locations of the six scenarios. 1: Finnmark, 2: Greenland I, 3: Greenland II, 4: Svalbard, 5: Kara Strait, 6: Varandey (see Table 1 for details). The model area of the SINMOD hydrodynamic model is shown in dashed outline

The SINMOD model area used to generate the hydrodynamic data for this study is shown in dashed outline in Fig. 1. The model area has a spatial resolution of 4 × 4 km, and the dataset produced has a temporal resolution of 2 h. Boundary conditions were taken from a larger model domain, at 20 × 20 km resolution. A total of 8 tidal components were imposed by specifying the various components at the open boundaries of the large-scale model. Tidal data were taken from TPXO 6.2 model of global ocean tides (Egbert et al. 1994).^1^

For the present climate simulation (2009–2012), atmospheric data from the ERA-Interim Reanalysis (Dee et al. 2011) have been used. For the climate change case (2050–2053), the atmospheric forcing fields come from a regional model system run by the Max Planck Institute, REMO (Keup-Thiel et al. 2006), and is based on the IPCC’s A1B scenario (Nakicenovic and Swart 2000). This model is configured to cover the model domain of SINMOD and has a grid resolution of approximately 0.22 degrees.

The ice model in SINMOD is a Hibler formulation (Hibler 1979), and has two state variables: average ice thickness in a grid cell, *h*, and the fraction of a grid cell covered by ice, *A*. The remaining fraction, 1 − *A*, is open water. The equation solver uses the elastic–viscous–plastic mechanism as described by Hunke and Dukowicz (1997).

### The OSCAR oil spill model

For the oil spill simulations in this paper, we have used OSCAR, which is a fully three-dimensional oil spill trajectory model for predicting the transport, fate and effects of released oil. The model accounts for weathering, the physical and chemical processes affecting oil at sea, as well as biodegradation. The development of models for these processes is strongly coupled with laboratory and field activities at SINTEF, on the transport, fate and effects of oil and oil components in the marine environment (Brandvik et al. 2013; Johansen et al. 2003, 2013, 2015).

The OSCAR model computes surface spreading of oil, slick transport, entrainment into the water column, evaporation, emulsification and shore interactions to determine oil drift and fate at the surface. In the water column, horizontal and vertical transport by currents, dissolution, adsorption and settling are simulated. The different solubility, volatility and aquatic toxicity of oil components are accounted for by representing oil in terms of 25 pseudo-components (Reed et al. 2000), which represent groups of chemicals with similar physical and chemical properties. By modelling the fate of individual pseudo-components, changes in oil composition due to evaporation, dissolution and biodegradation are accounted for. There is a biodegradation rate for each of the pseudo-components for the dissolved water fraction, droplet water fraction, surface and sediments (Brakstad and Faksness 2000).

OSCAR uses a Lagrangian particle transport model, where the release is represented by numerical particles (Reed et al. 2000). Each numerical particle is transported individually through the flow field. Buoyancy and sinking of oil droplets due to density differences or oil mineral aggregates are also included. Required inputs to the OSCAR model are currents, wind and ice (if relevant). The chemical composition of the released oil is also an essential part of the input to OSCAR.

### Scenarios and locations

Six scenarios were selected to be used as case studies. Details of the scenarios are provided in Table 1. We would like to stress that these scenarios are fictitious, and were made up to show how the footprint of an oil spill might differ under a climate change scenario. These scenarios are not meant to represent the most likely oil spill scenarios in the Arctic, and do not take into account expected changes in activities and shipping routes between the present and the future.

**Table 1:** Scenario parameters for the six case studies

	Finnmark	Greenland I	Greenland II	Svalbard	Kara Strait	Varandey
Longitude	26.75E	17.42W	14.70W	19.70E	58.60E	58.15E
Latitude	71.30N	78.68N	79.13N	76.73N	70.46N	69.05N
Release diameter (m)	0.6604	0.6604	0.6604	–	–	–
Release rate (mt/h)	208.3	208.3	208.3	66.6	66.6	66.6
Release duration (h)	72	72	72	3	3	3
Total amount (mt)	15 000	15 000	15 000	200	200	200
Release depth (m)	253	310	41	0	0	14
Sea depth (m)	254	311	42	155	15	15
Oil type	Statfjord C	Statfjord C	Statfjord C	Marine diesel	Marine diesel	Russian export crude
Simulation length (d)	15	15	15	15	15	15

The first scenario (Finnmark) is included because the release area has fully open water throughout the year, in both the present climate (2009–2012) and the future (2050–2053). It will serve as a “control scenario”, to compare to the five other scenarios, where there is a change in ice cover from present to future. The Finnmark scenario is a well blowout, with a release rate of 5000 metric tons per day, which is a little less than what was seen in the Deepwater Horizon oil spill (McNutt et al. 2012). The duration of 72 h is relatively short, and is representative of a scenario in which the well is successfully capped in short order.

The next two scenarios (Greenland I and Greenland II) are also well blowouts, with all parameters except location and depth equal to the Finnmark scenario. The difference between the two Greenland scenarios is the location, i.e. the depth and distance from the coast. We have selected to use the properties of crude oil from the Statfjord C field for the modelling of the three well blowouts. The Statfjord C Blend crude oil is regarded as a paraffinic medium crude oil with a density of 0.834 g/ml (API gravity 38). The fresh oil has a medium content of wax (4.1% by weight) and low asphaltenes (0.09% by weight) compared with other crude oils in the Norwegian sector. The oil exhibits a medium evaporative loss and forms relatively stable water-in-oil emulsions with high water content (approximately 80%).

The next two scenarios (Svalbard and Kara Strait) are shipping accidents, where we consider a surface spill of 200 metric tons of marine diesel, over a period of 3 h. For the two shipping accident scenarios, we have selected to use a marine diesel, which has a density of 0.843 g/ml (API Gravity 36.4), with a low pour-point (lower than − 24°C). The diesel oil will not emulsify and form stable water-in-oil emulsions and minor water uptake is expected. Both the fresh oil and its evaporated residues exhibit low viscosities.

The final scenario (Varandey) is a pipeline leak, near the Varandey oil export terminal, located 22 km off the coast, in a shallow area to the west of the Kara Strait. Two hundred metric tons of oil are assumed to leak out over a period of 3 h, from a depth of 14 m (1 m above the sea bed). Here we have chosen a Russian Export crude oil for the modelling. It is a medium density oil, at 0.871 g/ml (API gravity 31). The oil exhibits features of an intermediate between an asphaltenic and a more paraffinic oil, due to the relatively high content of asphaltenes (1% by weight). The oil exhibits a low to medium evaporative loss, and the oil will easily emulsify and form stable water-in-oil emulsions with high viscosities and high water content (70–80% by volume). This oil is expected to be dispersible with application of chemical dispersants.

### Ice cover

**Fig. 2:**
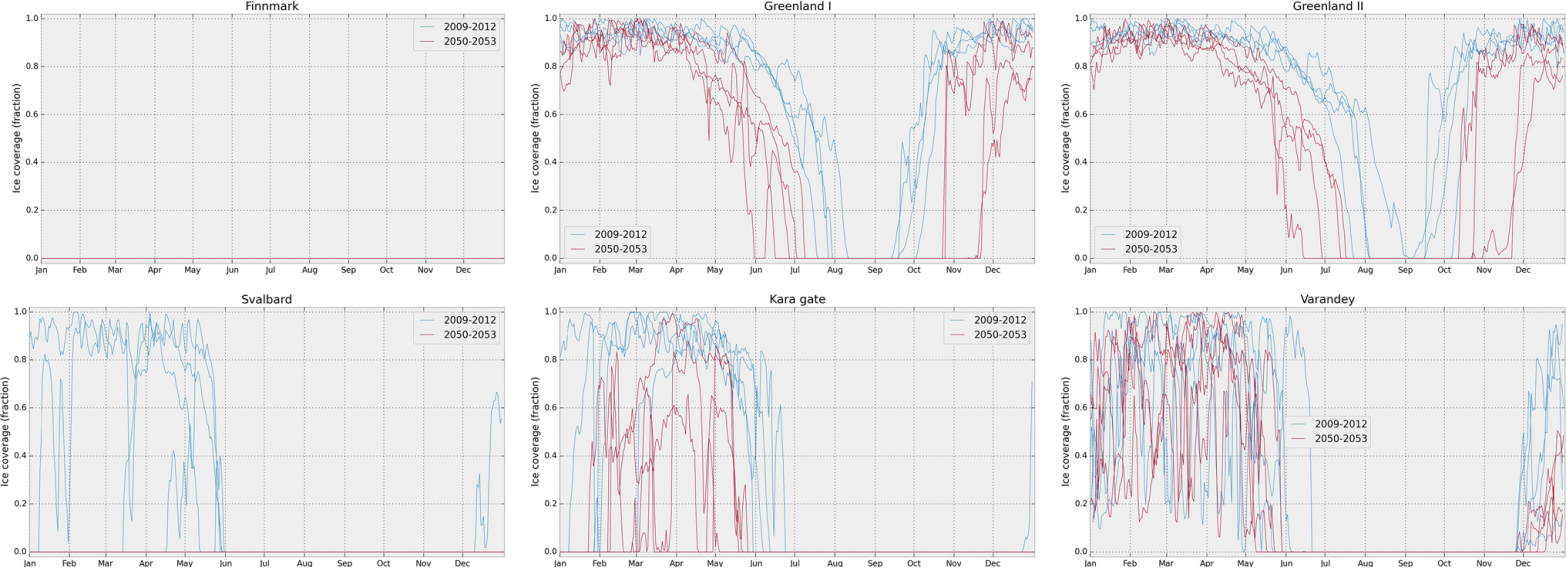
Ice coverage (fraction) at each release location, as a function of time of year, shown for the present and the future separately. Note that ice coverage is always 0 in the Finnmark scenario, and 0 in the future for the Svalbard scenario

In Fig. 2, the ice coverage (fraction of the sea surface covered by ice) is shown, for each of the six release locations. In the Finnmark scenario, there is fully open water both in the present and the future, while in the Svalbard scenario, there is quite high ice cover for several months of the year in the present, but always open water in the future. For the two Greenland scenarios, the change in ice cover can be clearly seen as a longer season of open water in the future scenarios. For the Kara scenario, there is clearly a winter season also in the future data, but it is shorter, and with less ice cover on average. For the Varandey scenario, the picture is less clear, with highly variable ice cover in both the present and the future, but on average there is somewhat less ice in the future. As a rule of thumb, a surface oil slick is considered to move as if in completely open water for ice coverage less than 30%, and as if in full ice cover if the ice coverage is above 70%. The current and wind data show less clear differences between present and future. Some information on the wind and current data can be found in the form of wind/current roses in Figs. S1–S4 in Supplementary Material.

### Ensemble simulations

Even in a future with a warmer climate, there is no guarantee that any given day, or even a given year, will be warmer than in the present. Rather, global average temperatures will be higher, with potential changes in weather patterns, prevailing winds, ice cover, etc. As climate change is a statistical phenomenon, any single simulation of an oil spill at a given time and location is not particularly relevant for this study. Instead, we are interested in how the distribution of probable outcomes will change with the changing climate.

Ensemble simulation methods are commonly used in the study of chaotic systems, such as the weather and the ocean. In this paper, when referring to an ensemble, we mean a collection of oil spill simulations, where each simulation has used different environmental forcing data. The environmental data are available as two archives, one historical hindcast archive, covering 2009–2012 (and some months into 2013), and one climate forecast archive, covering 2050–2053 (and some months into 2054). The variation in environmental data is achieved through varying the start date of the simulation, and selecting the corresponding data (current, wind, ice cover and water temperature) from the two archives. In a real oil spill at a given location, the timing will determine the currents, winds, waves and other meteorological conditions, which together with the release parameters determine the transport and fate of the spill. Hence, by carrying out large ensembles of oil spill simulations with different start times, we sample from the distribution of possible predicted outcomes for that location.

We have performed ensemble simulations for six different scenarios (see Table 1). For each scenario, we started one simulated oil spill every 6 h for 2 × 4 years, in total 11 688 simulations per scenario. Each simulation was set up as an individual OSCAR scenario, and took approximately 6 min to run, for a total of about 7000 CPU hours. The simulations were carried out on a 32-core compute node, using the linux version of OSCAR.

## Results

The simulation results from the OSCAR model include four-dimensional (*x*, *y*, *z*, *t*) concentration fields giving concentration per pseudo-component for droplets and dissolved chemicals, as well as three-dimensional (*x*, *y*, *t*) grids for oil on the sea surface, on the shore and in the sediments.

Furthermore, some aggregated quantities from each simulation are available as time series. These include amounts of evaporated oil, oil on the sea surface, submerged oil, oil on the shore, oil in the sediment and amount of oil which has been biodegraded. These six quantities make up the mass balance, and give information about the fraction of the total released mass which is found in any given “environmental compartment”. During the development of a spill, oil can move between compartments: Oil on the surface can be mixed down by waves and submerged, submerged oil can resurface, stranded oil can be washed out to sea, etc. The exception is that evaporated or biodegraded oil is removed from the simulation. Note that oil which is trapped at the ice/water interface under sea ice is included in the “surface” compartment.

**Fig. 3:**
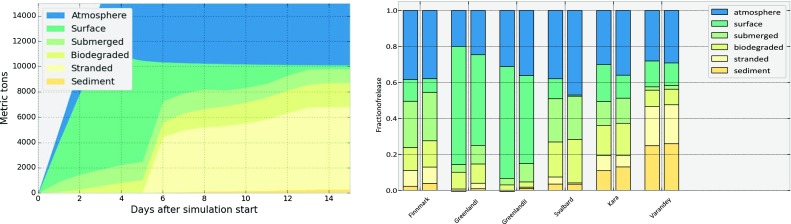
To the left, an example of the development of the mass balance for a simulation with significant stranding after about 5 days. To the right, average mass balances for all six scenarios. For each scenario, the left column represents the present (2009–2012), and the right column represents the future (2050–2053)

In Fig. 3 (left), an example of the development of the mass balance is shown for one's realisation of the Finnmark scenario. We see how the oil is released at a constant rate over 3 days, reaching a total of 15 000 metric tons. Evaporation and biodegradation are continuously ongoing processes, while we note that a significant fraction of the oil hits shore after about 5 days. At the end of the 15-day simulation, 33% of the oil has evaporated, 2% is at the surface, 7% is submerged, 13% has been biodegraded, 43% has stranded and 2% has ended up in the sediments. It should be mentioned that this simulation is the worst case from the Finnmark scenario, with respect to amount of stranded oil.

The goal of this study is to investigate how the footprint of a “typical” oil spill will change with a warmer climate. In order to obtain statistical results, we have chosen to consider the amount of oil in the six environmental compartments only, e.g. considering the amount of oil on the shore, without reference to exactly where that oil has beached. For the further analysis in this study, we will only use the data at the end of each simulation, corresponding to day 15 in the left panel of Fig. 3.

Furthermore, in Fig. 3 (right), the average amount in each compartment is shown separately for each of the six scenarios, and for the present and the future. We note that in all cases, there is some difference between the present and the future, but the nature and magnitude of the change differs for each scenario.

**Fig. 4:**
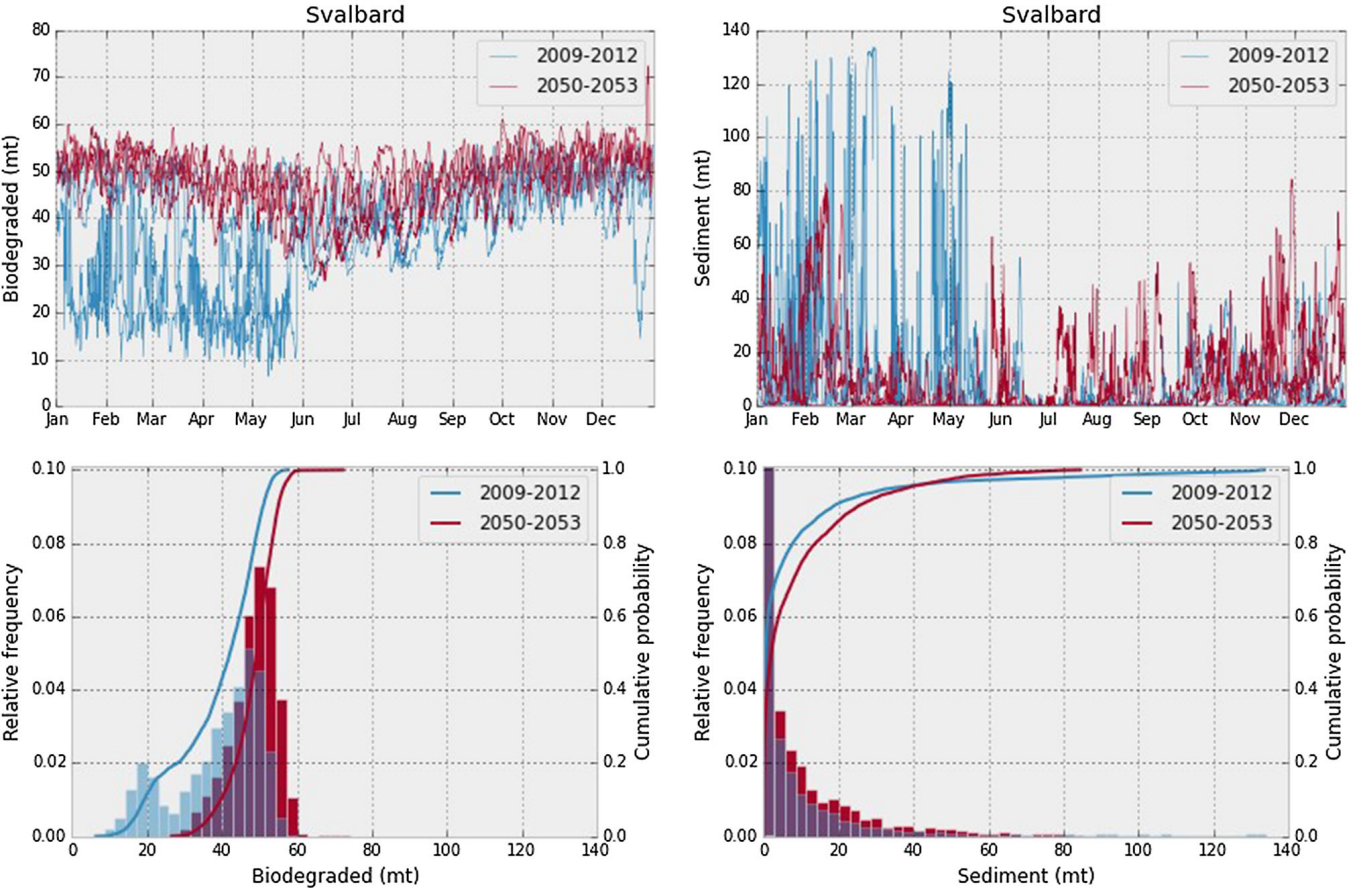
Top row: Time series showing amount of biodegraded oil (left) and amount of oil in the sediments (right), at the end of each simulation, as a function of start date of the simulation, for the Svalbard scenario. Present and future shown separately. Bottom row: Histogram and empirical distribution for the time series above, with present and future shown separately. Note that the axis has been truncated for visibility in the sediment plot. The height of the first column is 0.24 for the present and 0.19 for the future

In addition to average values, another way of presenting the results of an ensemble of simulation is as a time series showing the amount of oil in an environmental compartment at the end of a simulation, as a function of the start time of that simulation. In Fig. 4 (top row), this is shown for amount of biodegraded oil and amount of oil in the sediment, for the Svalbard scenario. From Fig. 2, we see that in the future scenarios (2050–2053), there is always open water at the release location of the Svalbard case, while in the present scenarios, there is high ice cover roughly from January to the end of May, with some variation among the years. Clearly, the presence of ice coincides with the onset of a completely different behaviour in the time series for the present shown in Fig. 4. We note also that the behaviour in the ice-free season seems quite similar for the present and the future.

As was recently shown (Nordam et al. 2016), the amounts of oil in the different compartments exhibit different probability distributions. In particular, the amount of stranded oil and oil in the sediment seem to follow some variety of a fat-tailed distribution. Consequently, average values may be of little use, or even misleading, and applying intuition from Gaussian distributions can cause severe underestimation of the probable worst-case scenario. As an extreme example, consider the amount of stranded oil in the present time in the Greenland II scenario: The 95-percentile (the amount which is such that in 95% of cases, the amount is less than this) is 1 metric ton, the 99-percentile is 83 tons and the 99.9-percentile is 1317 tons. For a Gaussian distribution of the same mean and standard deviation (*µ* = 9.47 tons and *σ* = 98.8 tons), the 99- and 99.9-percentile would be, respectively, 1.4 times and 1.8 times the 95-percentile.

Since the distributions vary, a more complete way of displaying the results of the ensemble simulation is to consider the empirical distribution of each endpoint. In Fig. 4 (bottom row), the distributions for amount of biodegraded oil, and amount of oil in the sediments, are shown for the Svalbard scenario, both as a histogram and as an empirical cumulative probability distribution, and separately for the present and the future. Note how the amount of biodegraded oil exhibits a bi-modal distribution in the present, due to a different behaviour when there is ice, and note also how the two endpoints (biodegraded and sediment) display completely different distributions.

**Fig. 5:**
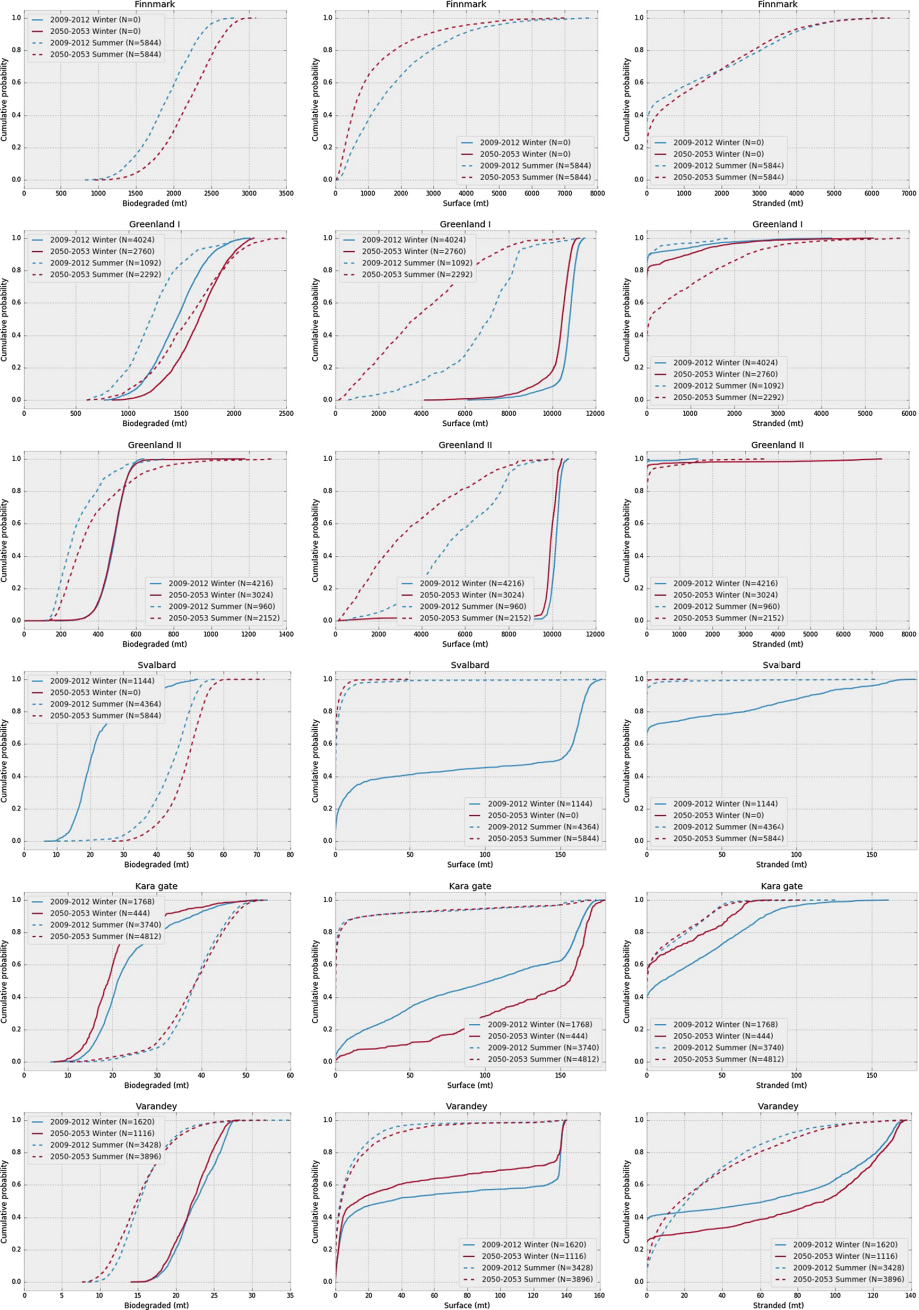
Empirical distribution of the amount of biodegraded oil, amount of oil on the surface and amount of stranded oil, 15 days after the start of the release, for all scenarios. Simulations are classified as Winter (70% ice cover or higher) or Summer (30% ice cover or less). The number of simulations in each category is given in the legends. Note that in some scenarios the ice cover never exceeds 70%

In Table 2, we show the mean, the standard deviation and the 95-percentile for the amount of biodegraded oil, oil on the surface and stranded oil, as well as the total area of the surface slick, for all six scenarios, and separately for the present and the future. From Fig. 4, we note that the difference between summer and winter in the present seems to be much larger than the difference between the present and the future. In order to investigate the effect of ice cover in determining the seasons, we can categorise each simulation in the ensembles as “winter” if the ice coverage is above 70% at the release location at the start of the release, and as “summer” if the ice coverage is below 30%. This split will then allow us to construct the empirical distributions for the summer and winter data separately, and compare between present and future. In Fig. 5, the empirical distributions are shown for amount of biodegraded, surface and stranded oil in all scenarios, split into winter and summer as described above. Note that in the Finnmark scenario, there is no ice, neither in the present or in the future, and in the Svalbard scenario, there is no ice in the future scenarios, and consequently no points are classified as winter.

**Table 2:** Average (Avg.), standard deviation (Std.) and 95-percentile (95%) of amounts of biodegraded oil, oil on the surface and stranded oil (in metric tons), as well as surface slick area (in square kilometres), with present and future shown separately for each scenario

	Biodegraded (mt)			Surface (mt)			Stranded (mt)			Total area (km^2^)		
	Avg.	Std.	95%	Avg.	Std.	95%	Avg.	Std.	95%	Avg.	Std.	95%
Finnmark (present)	1897	365.8	2461	1815	1450	4732	1325	1608	4445	870.3	936.3	2765
Finnmark (future)	2194	377	2759	1136	1202	3831	1367	1509	4326	712.3	813.5	2121
Greenland I (present)	1392	284.4	1877	9850	1910	11 190	111.3	443.6	941.3	216.2	545.8	1392
Greenland I (future)	1601	311	2084	7578	3456	10 870	459.6	917.4	2499	443.3	849.7	2201
Greenland II (present)	441.2	103.3	572.4	9315	2016	10 500	9.469	98.79	1.005	316.1	823.9	2165
Greenland II (future)	432.8	137.1	615.1	7321	3516	10 330	100.4	618.8	164.4	722.2	1242	3340
Svalbard (present)	38.84	11.46	52.48	22.08	51.42	162.9	7.879	28.04	76.23	2.638	6.256	12.94
Svalbard (future)	48.3	5.949	56.32	1.093	2.932	5.878	0.062	0.611	0.18	1.279	2.795	7.341
Kara gate (present)	33.38	10.08	47.45	41.2	61.66	163.5	16.75	25.66	70.94	2.763	7.986	13.29
Kara gate (future)	35.86	9.447	48.77	25.53	51.6	161.9	12.38	20.08	55.21	2.779	6.968	13.29
Varandey (present)	18.27	4.489	26.37	28.7	47.37	136.7	43.41	44.28	128.2	5.974	15.28	27.56
Varandey (future)	17.25	4.437	25	25.07	42.23	136.4	43.29	44.59	128.2	7.413	18.51	35.88

Finally, in order to give a simple estimate of the relative impact of the various environmental inputs, on the amount of oil that ends up in the different environmental compartments, we have calculated the normalised cross-correlations. The normalised cross-correlation of two discrete series, *x*_*i*_ and *y*_*i*_, each with *N* elements, is given by

1 $$ \frac{1}{N}\sum\limits_{i} {\frac{{(x_{i} - \mu_{x} )(y_{i} - \mu_{y} )}}{{\sigma_{x} \sigma_{y} }}} , $$

where *µ*_*x*_ and *σ*_*x*_ are the mean and standard deviation of *x*_*i*_, and *µ*_*y*_ and *σ*_*y*_ are the same for *y*_*i*_.

**Fig. 6:**
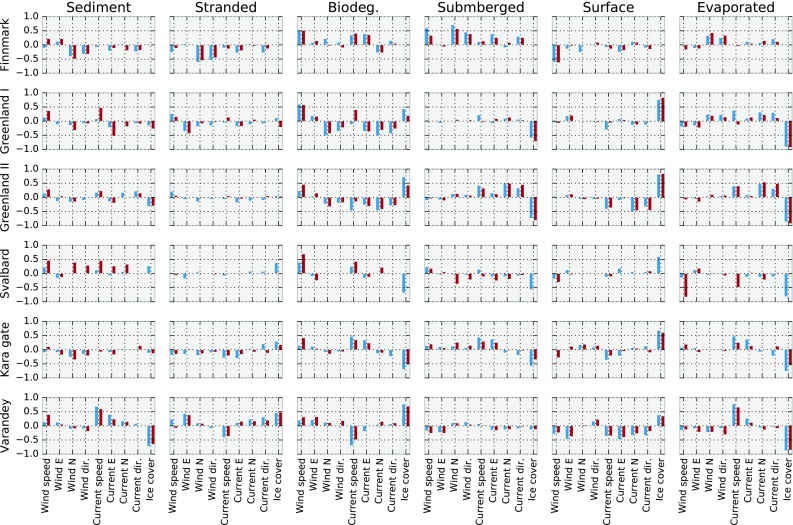
Normalised cross-correlation of amount in the six environmental compartments, with five environmental inputs: wind speed, wind direction, current speed, current direction and ice cover. Present (2009–2012) shown in blue, future (2050–2053) shown in red. All environmental inputs are averaged over the 15-day simulation period, and cross-correlations are calculated by matching the time series of simulation results with the averaged input from each simulation

The normalised cross-correlations are shown in Fig. 6. In calculating the correlations, the environmental inputs have been averaged over the 15 days of each simulation. Thus, if the cross-correlation between, e.g. the amount of stranded oil and the ice cover is calculated, then *x*_*i*_ is the amount of stranded oil at the end of simulation *i*, and *y*_*i*_ is the average ice cover at the release location during the 15 days of simulation *i*. Note again that for the Finnmark scenario, the ice cover is always 0, and for the Svalbard scenario, the ice cover is always 0 in the future, and hence the correlations with ice cover will be 0 in these cases.

Additional results from the complete ensembles of simulations are shown in Figs. S5–S10 in Supplementary Material.

## Discussion

From the results of the ensemble simulations, we find, not unexpectedly, that the presence of high ice cover will significantly alter the dynamics of an oil spill. Interestingly, however, the presence of ice does not only change the average values of oil in each environmental compartment. Instead, the probability distributions for amount of oil in the different compartments are modified, as can be seen from the empirical distributions shown in Fig. 5. This is especially clear for the amount of oil on the surface, which in turn is very relevant for amount of stranded oil, and potential for contact with birds or mammals.

For the amount of biodegraded oil, and to some degree the amount of oil on the surface, there is a clear difference between summer and winter, but little change from present to future. This indicates that the main reason for any differences between present and future is the longer ice-free season in the future. For the amount of stranded oil, the picture is less clear. This is however not entirely unexpected, since the amount of stranded oil seems to follow a more fat-tailed distribution (Nordam et al. 2016), and accurately estimating the parameters of a fat-tailed distribution requires considerably more data than estimating the parameters of a more “normal” (Gaussian-like) distribution. Considering the Varandey and Svalbard scenarios, and to some degree the Kara scenario, there is a clear increase in, e.g. the 95-percentile of amount of stranded oil in the winter season. For the two Greenland scenarios, however, the 95-percentile is essentially unchanged from summer to winter in the present case, and decreasing from summer to winter in the future.

From Fig. 6, we see that in many cases ice cover shows the strongest correlation with the different environmental endpoints. For example, in each case where there is ice, there is a strong, negative correlation between amount of evaporated oil and ice cover, meaning that a higher ice cover on average means less evaporation, which is to be expected. As is also to be expected, there is a strong negative correlation between ice cover and amount of submerged oil, and a strong positive correlation between ice and amount of oil at the surface (except in the Varandey case, where both correlations are less strong), the reason being that the ice prevents waves from submerging the oil. Recall that oil trapped at the ice–water interface is considered to be at the surface.

Perhaps more surprising is that amount of biodegraded oil is positively correlated with ice cover in some cases, and negatively in others. We also note that except in the Varandey case, ice cover does not show a particularly strong correlation to amount of oil in the sediment, which is somewhat surprising, given that open water and waves are required to submerge the oil and bring it into contact with the sea floor. A more detailed study of the spatial distribution of ice in each individual simulation would be required to adequately explain these results.

We note that even if ice cover does not always have the strongest correlations with the environmental endpoints, it still seems to work very well as a criterion for separating simulations into winter and summer dynamics. For example, for the amount of oil on the surface in the Varandey scenario, ice cover does not stand out among the correlations in Fig. 6, yet the split on ice cover produces an empirical distribution for the summer that is quite similar between present and future, and quite distinct from the winter distributions, which are again very similar between present and future (see Fig. 5).

Finally, it should be noted that it would have been ideal to have available longer time series of environmental data than the 2 × 4 years used here. As was shown by Nordam et al. (2016), the variations in ensemble results between years can be quite significant. Furthermore, phenomena like the Arctic Oscillation (Rigor et al. 2002) can affect the ice cover on a time scale of one or more years, leading to a possible bias in the results.

## Conclusions

In general, there is a change in the fate of a “typical” oil spill from the present to the warmer future considered here. The change seems to mainly come from the fact that there is a longer open water season in the future. Furthermore, the nature of the change depends on the scenario considered. For the two Greenland cases, we see from Table 2 that there is a larger (in terms of area) surface slick, more oil on the shore and larger variations in the future. In the Svalbard scenario, on the other hand, there is a substantial decrease in expected amount of stranded oil and area of the surface slick, while for the Kara scenario, there is little change in either endpoint. While the specific geographic distribution of oil has not been considered in this study, it seems reasonable to assume that the exact effect of a change in, e.g. ice cover, will be highly dependent on local conditions. For example, for a near-shore release, the ice can act to either shield the coast or trap the oil along the coast, depending on the spatial distribution of ice.

We have demonstrated that ice cover is extremely important for the prediction of the probable fate of an Arctic oil spill. Not only does the presence of ice affect the average mass balance of an oil spill, but it also modifies the probability distributions associated with the different environmental compartments. When carrying out an Environmental Risk Assessment, it is essential to have an idea of the probability distributions involved, in order to design the ensemble of simulations in a way that will give statistically robust results. The integration of ice cover into oil spill trajectory models is still a developing field, and this study demonstrates the importance of continued research, given the significant impact ice cover has on the results of the oil spill simulations. The further development of high-resolution coupled ice-ocean models is also essential, as for example the amount of stranded oil is expected to be very sensitive to the local distribution of sea ice.

The probability of oil spill accidents in the Arctic is expected to increase in concert with increases in transport and resource exploration and extraction activities. The results reported here suggest that future oil spills in a warming climate will in some cases result in greater areal coverage and increased shoreline exposure, due to reduced ice coverage. These two considerations point towards an increase in environmental risk, defined as the probability of an event, in this case an oil spill, weighted by the magnitude of the resulting environmental injury. Furthermore, the study highlights the need to take ice into account in Environmental Risk Assessments for petroleum operations in the Arctic, as well as the need for further development of ice modelling, as well as oil-in-ice trajectory modelling. Finally, longer time series of environmental data, ideally 10 years or more, should be used for Environmental Risk Assessments in the Arctic.

## Electronic supplementary material

Below is the link to the electronic supplementary material.

Supplementary material 1 (PDF 9969 kb)

## Footnotes

1 See also http://volkov.oce.orst.edu/tides/global.html.
