# Supplementary material for: Impact of climate change and seasonal trends on the fate of Arctic oil spills
Source: Ambio. 2017 Oct 24;46(Suppl 3):442–52. doi: 10.1007/s13280-017-0961-3 (PMC5673877; doi:10.1007/s13280-017-0961-3)
Supplement: Supplementary file 1 — Supplementary material 1 (PDF 9969 kb) [file 13280_2017_961_MOESM1_ESM.pdf]

Ambio

Electronic Supplementary Material
This supplementary material has not been peer reviewed

Title: **Impact of Climate Change and Seasonal Trends on the Fate of Arctic Oil Spills**

Authors: Tor Nordam, Dorien A.E. Dunnebier, CJ Beegle-Krause, Mark Reed, Dag Slagstad

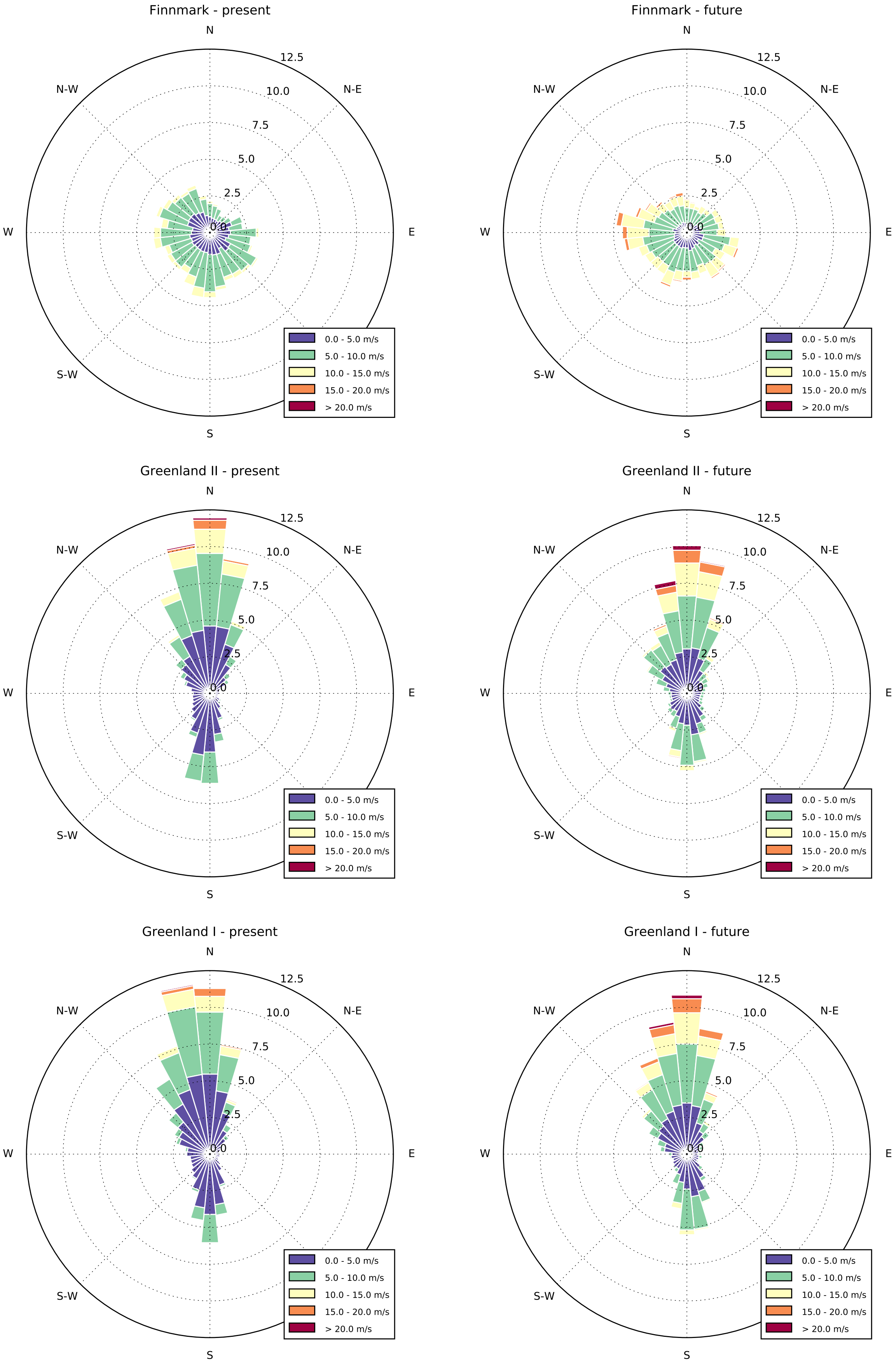

Figure S1: Wind roses showing comparison of wind conditions at the release point for each scenario, with present conditions on the left, and future conditions on the right. The length of each sector indicates the relative frequency of wind in that direction.

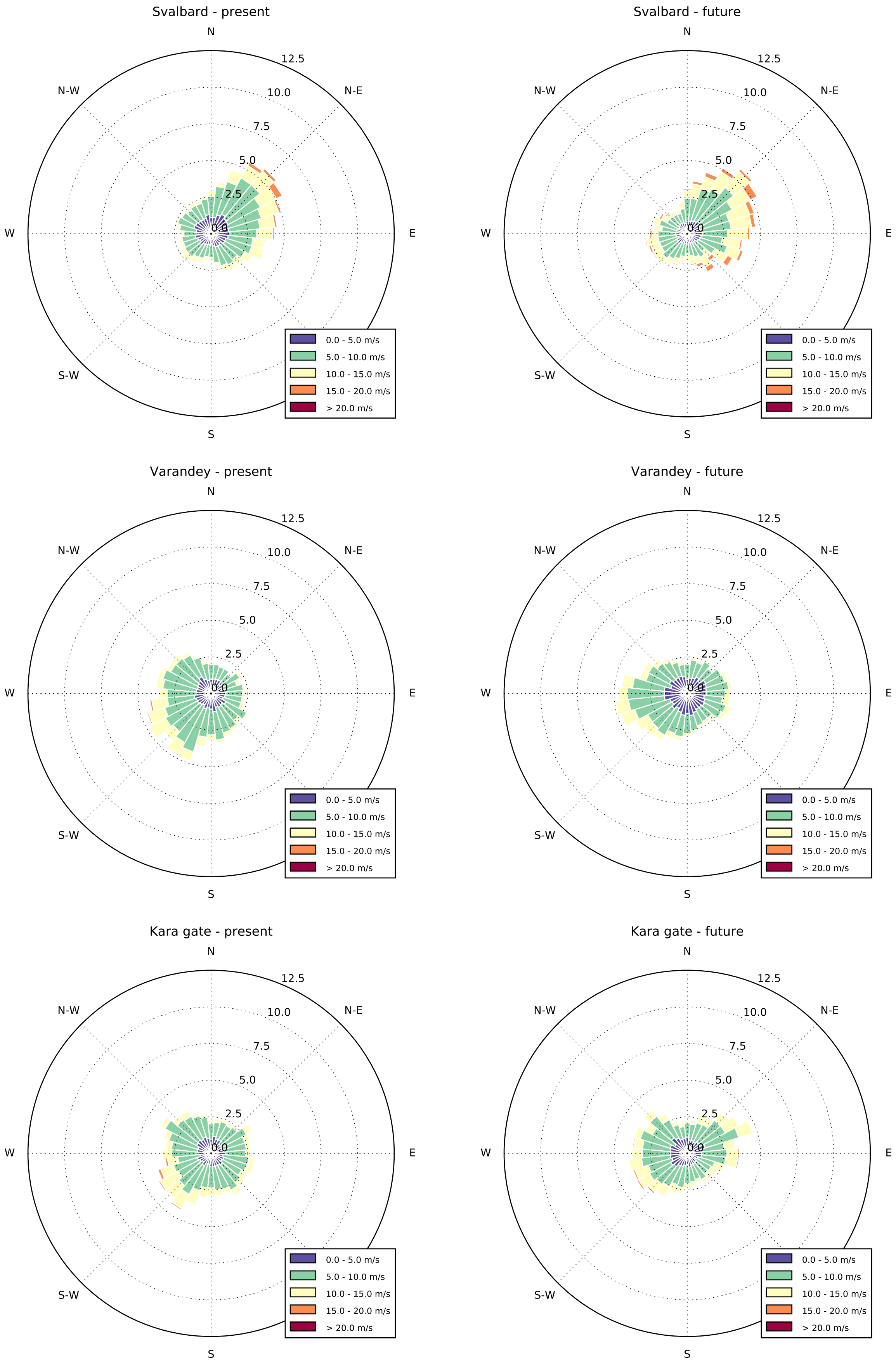

Figure S2: Wind roses showing comparison of wind conditions at the release point for each scenario, with present conditions on the left, and future conditions on the right. The length of each sector indicates the relative frequency of wind in that direction.

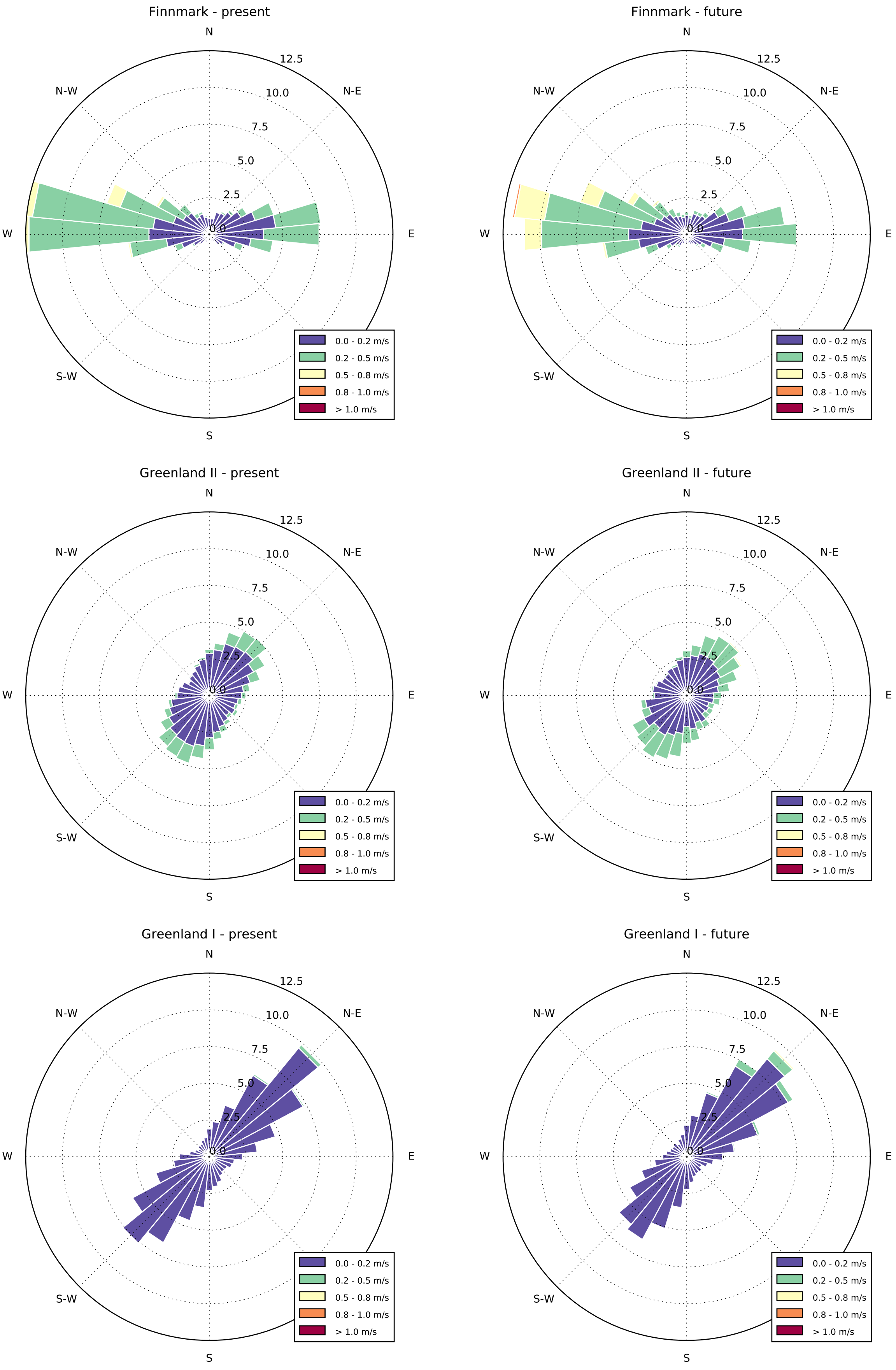

Figure S3: Current roses showing comparison of wind conditions at the release point for each scenario, with present conditions on the left, and future conditions on the right. The length of each sector indicates the relative frequency of current in that direction.

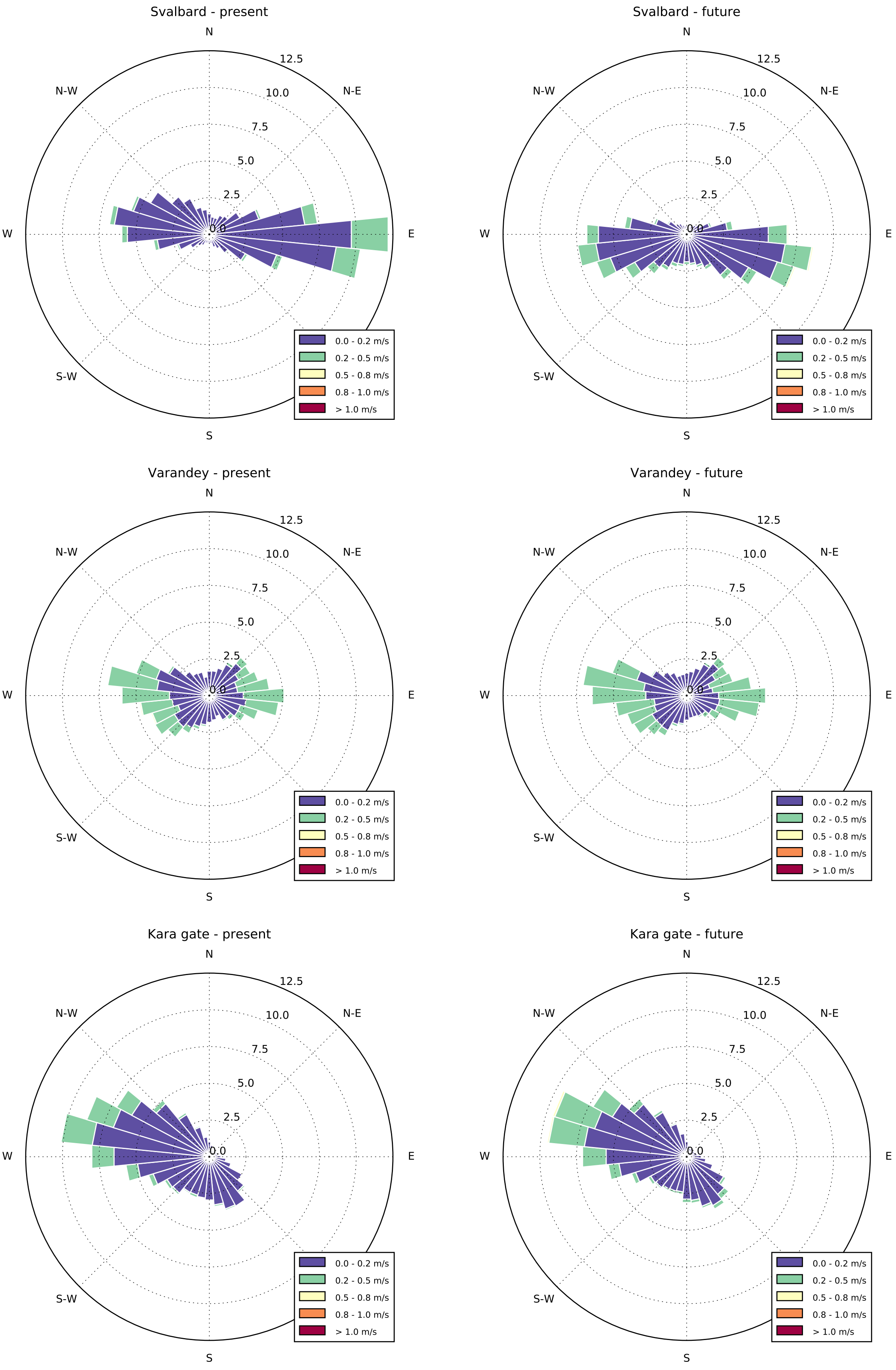

Figure S4: Current roses showing comparison of wind conditions at the release point for each scenario, with present conditions on the left, and future conditions on the right. The length of each sector indicates the relative frequency of current in that direction.

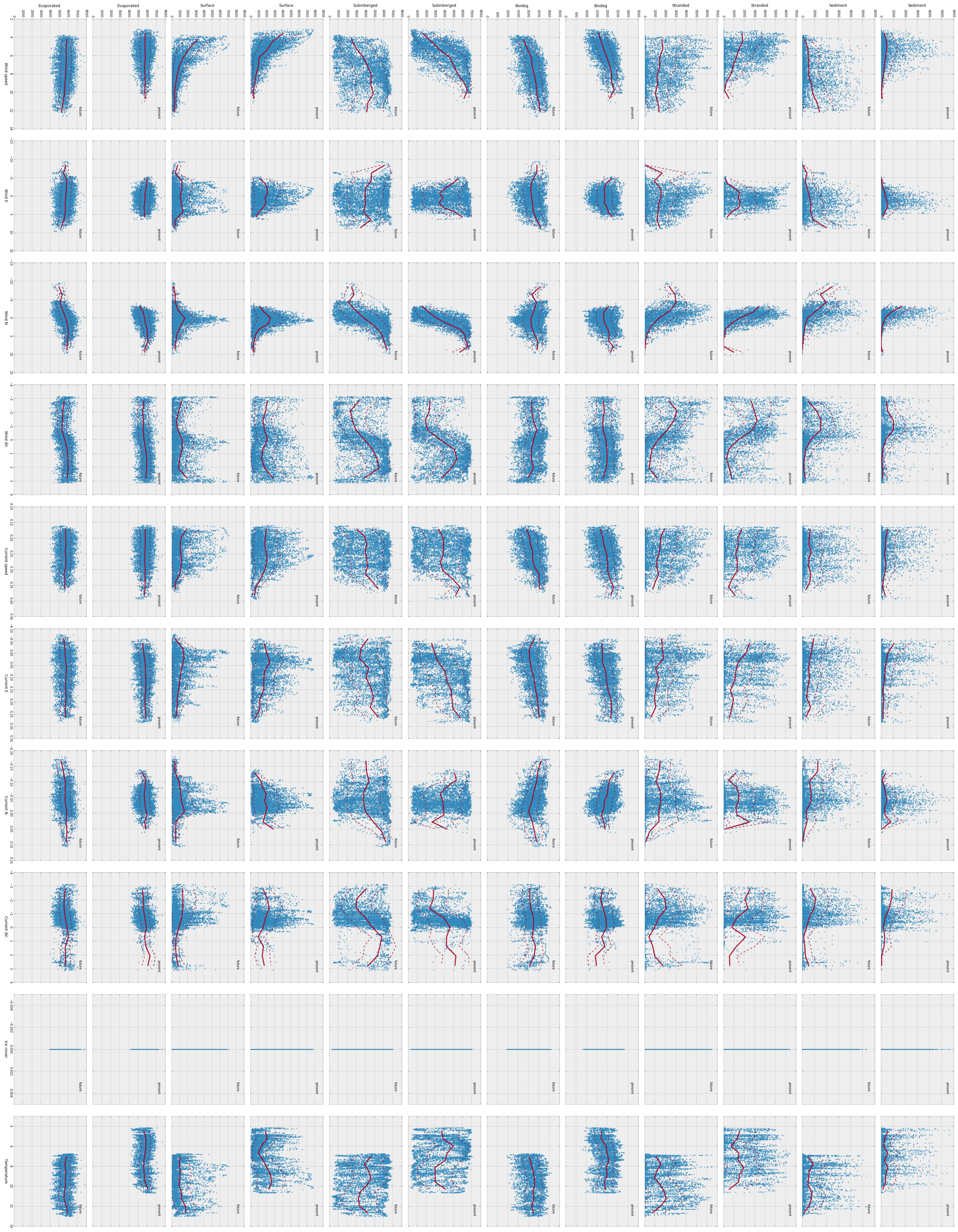

Figure S5: For the complete ensemble of simulations in the Finnmark scenario, the scatterplots show the amount (in metric tons) of oil in each compartment of the mass balance, at the end of the simulation, as a function of the average of each of the meteorological inputs during that simulation. The red line indicates the average, and the dashed red lines indicate one standard deviation in both directions.

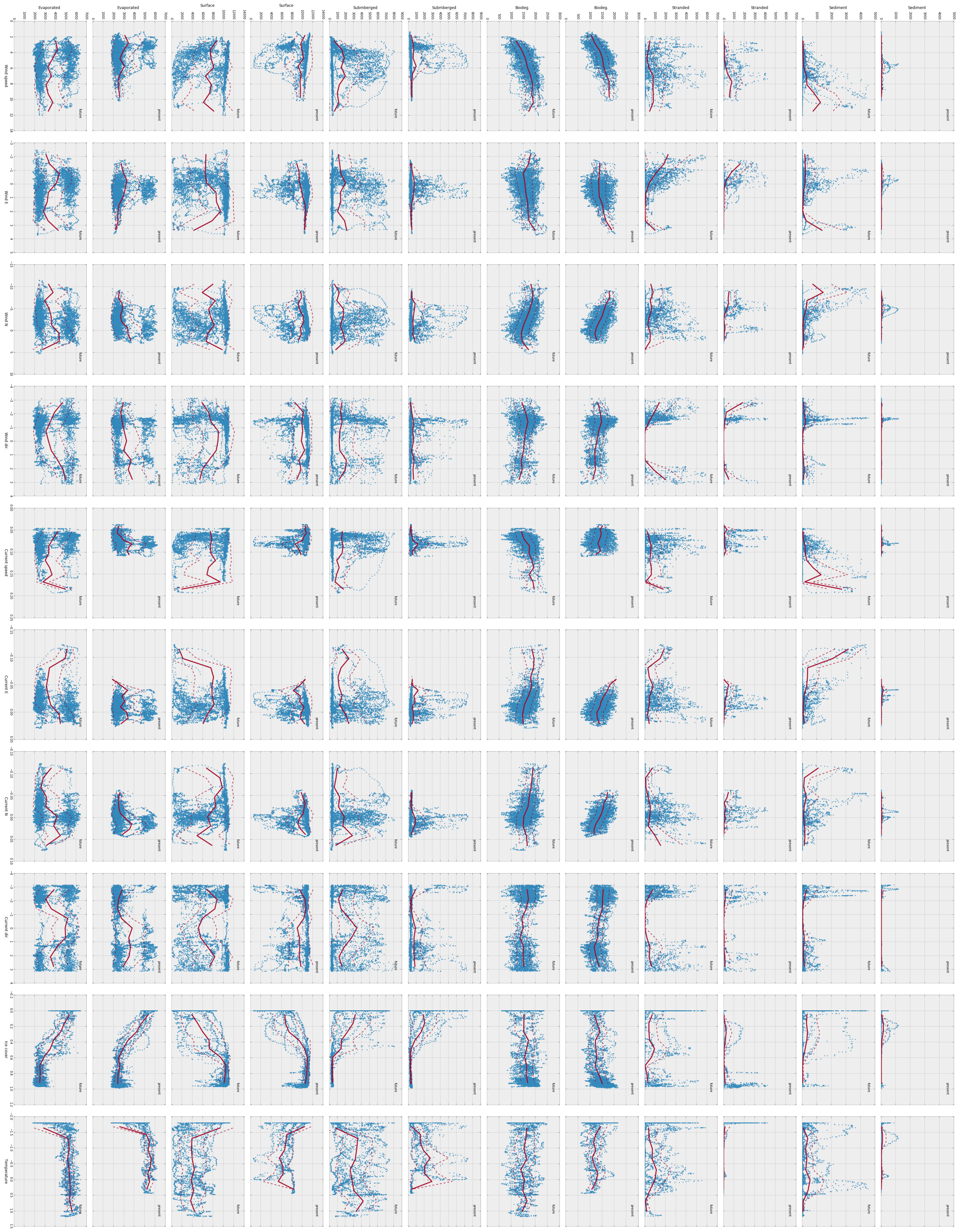

Figure S6: For the complete ensemble of simulations in the Greenland I scenario, the scatterplots show the amount (in metric tons) of oil in each compartment of the mass balance, at the end of the simulation, as a function of the average of each of the meteorological inputs during that simulation. The red line indicates the average, and the dashed red lines indicate one standard deviation in both directions.

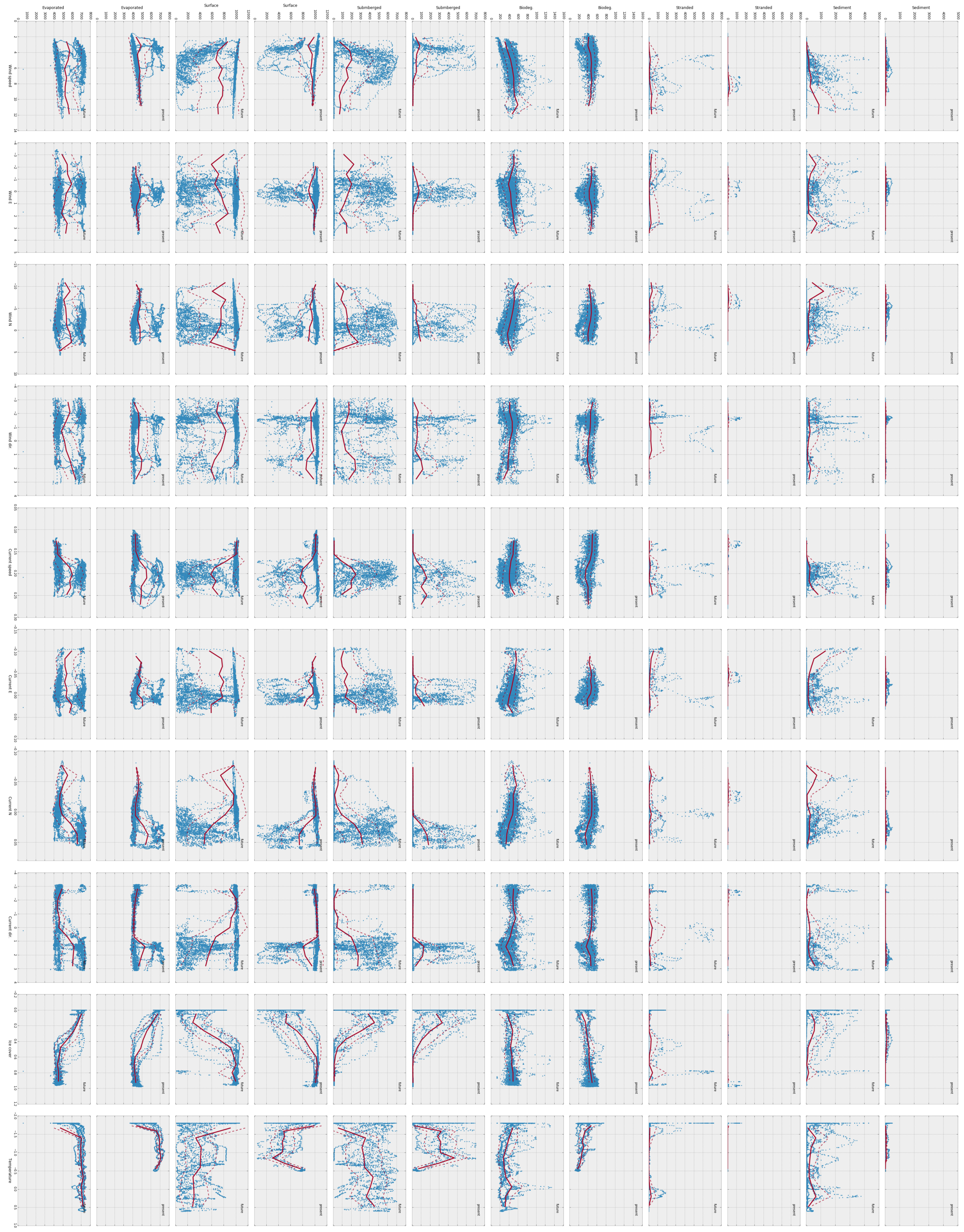

Figure S7: For the complete ensemble of simulations in the Greenland II scenario, the scatterplots show the amount (in metric tons) of oil in each compartment of the mass balance, at the end of the simulation, as a function of the average of each of the meteorological inputs during that simulation. The red line indicates the average, and the dashed red lines indicate one standard deviation in both directions.

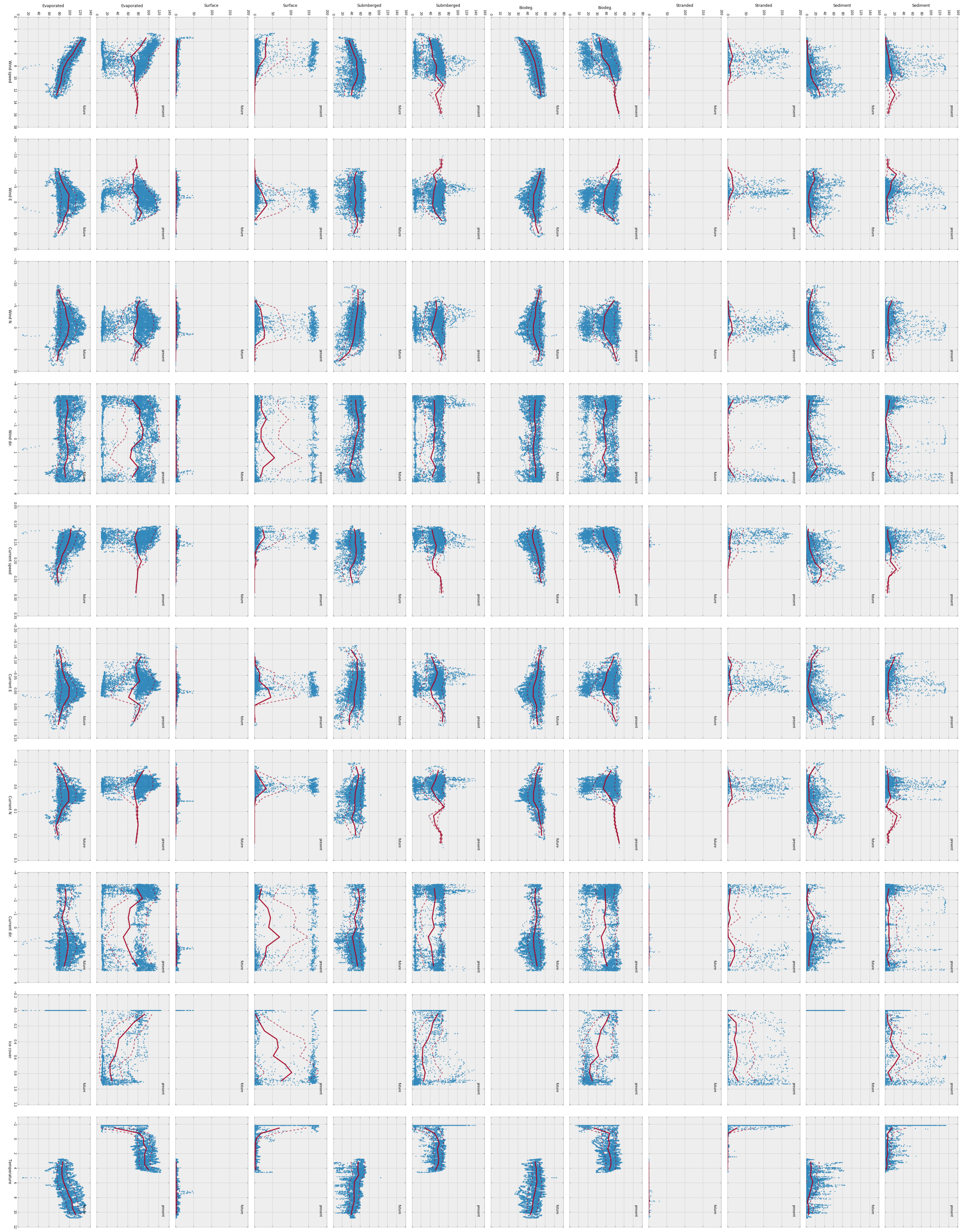

Figure S8: For the complete ensemble of simulations in the Svalbard scenario, the scatterplots show the amount (in metric tons) of oil in each compartment of the mass balance, at the end of the simulation, as a function of the average of each of the meteorological inputs during that simulation. The red line indicates the average, and the dashed red lines indicate one standard deviation in both directions.

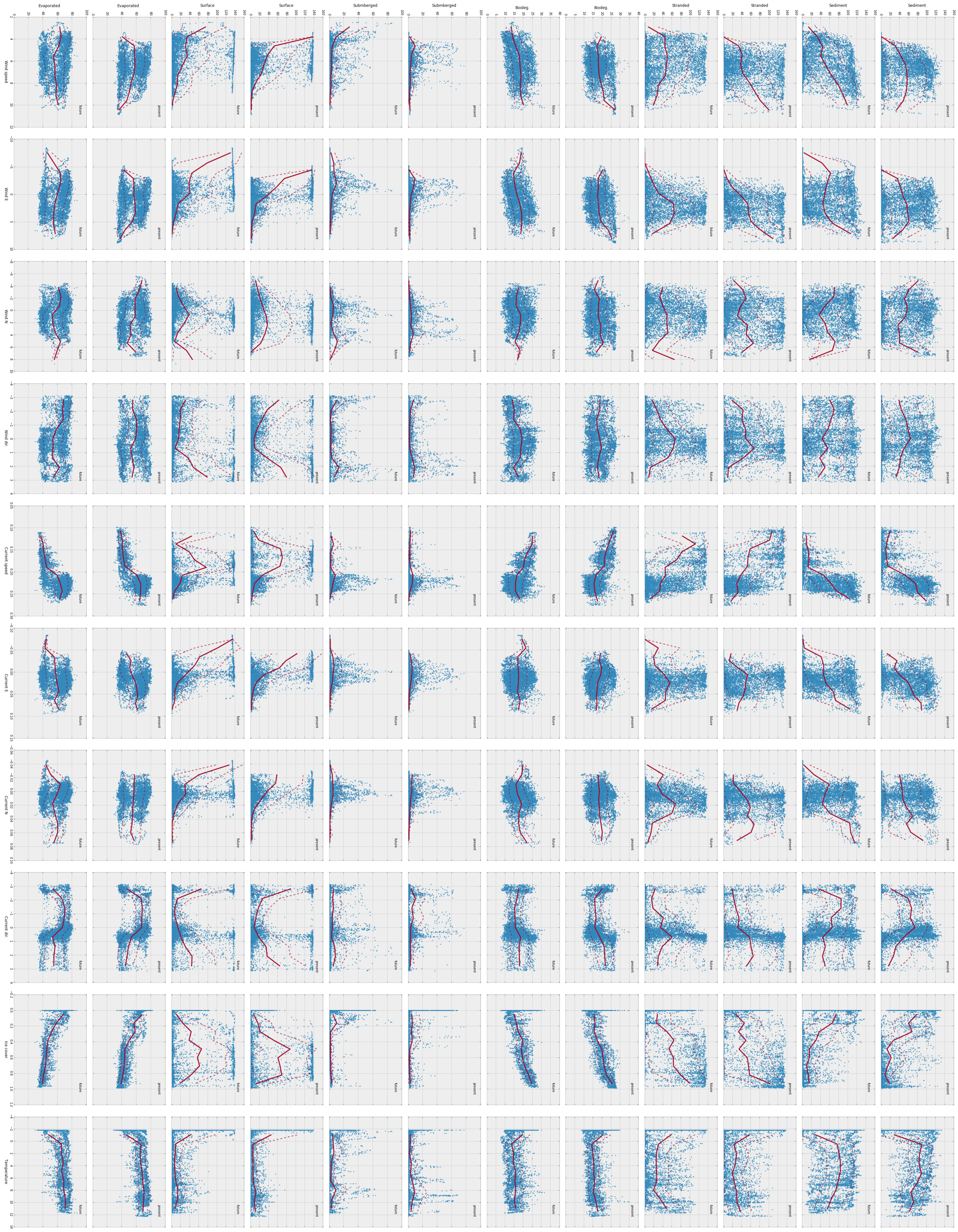

Figure S9: For the complete ensemble of simulations in the Varandey scenario, the scatterplots show the amount (in metric tons) of oil in each compartment of the mass balance, at the end of the simulation, as a function of the average of each of the meteorological inputs during that simulation. The red line indicates the average, and the dashed red lines indicate one standard deviation in both directions.

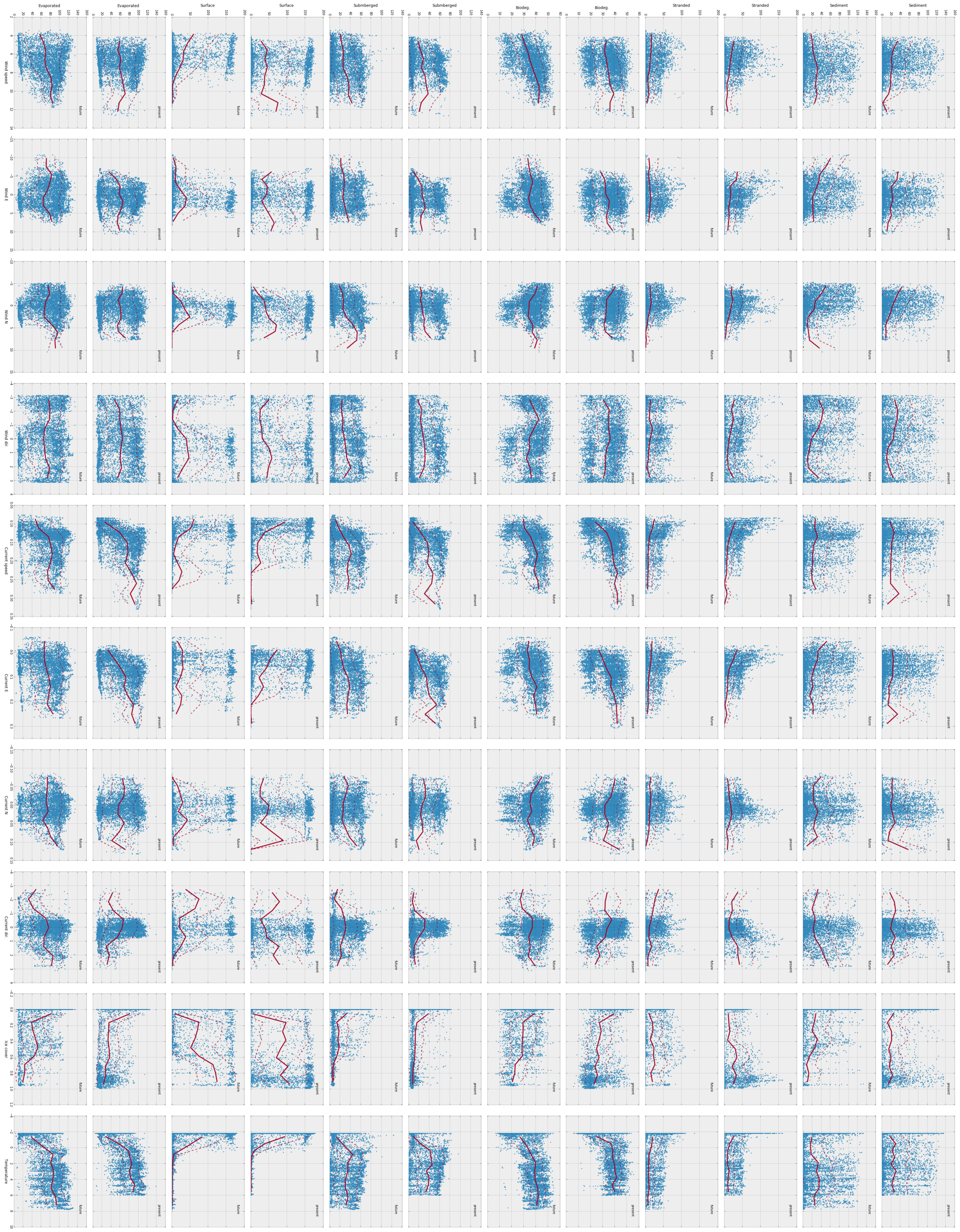

Figure S10: For the complete ensemble of simulations in the Kara scenario, the scatterplots show the amount (in metric tons) of oil in each compartment of the mass balance, at the end of the simulation, as a function of the average of each of the meteorological inputs during that simulation. The red line indicates the average, and the dashed red lines indicate one standard deviation in both directions.
